# Impervious cover is associated with culture-based morphotype diversity in urban stormwater runoff

**DOI:** 10.1128/spectrum.04051-25

**Published:** 2026-09-25

**Authors:** Toby He Qin

**Affiliations:** 1 East Chapel Hill High School 308376 , Chapel Hill, North Carolina, USA; Connecticut Agricultural Experiment Station, New Haven, Connecticut, USA

**Keywords:** urban stormwater runoff, microbial diversity, impervious cover, alpha diversity indices, predictive modeling, cross-validation, linear mixed-effect models, LMM

## Abstract

Urban stormwater runoff transports diverse biological material into receiving waters, but whether storm-by-storm culture-based diversity is associated with simple environmental variables is not well quantified. This study examined whether culture-based morphotype diversity in urban runoff was associated with impervious cover percentages and basic weather variables. Runoff was sampled during 25 storms at three Chapel Hill, NC, catchments representing green, residential, and commercial urban land-use patterns to obtain 75 site-storm samples. For each sample, colonies grown on nutrient agar were segmented into morphotypes from overhead images, and Shannon, Gini–Simpson, and Pielou evenness indices were computed from morphotype area fractions. Shannon diversity and Simpson diversity were consistently highest for the green space site, intermediate at the residential space site, and lowest for the commercial space site, while evenness showed the opposite pattern. Linear mixed-effect models with storm as a random effect identified land-use type, used here as a proxy for impervious cover, as the dominant predictor, whereas 24-h rainfall and daily mean air temperature showed weak associations. These results show a strong and repeatable association between impervious cover and culture-based runoff morphotype diversity. Because the assay relies on nutrient agar culturing and visible colony morphotypes and includes no non-storm controls, it should not be interpreted as a measure of total microbial diversity.

**IMPORTANCE:**

By sampling 25 storms in three contrasting urban catchments and using culture-based morphotypes on nutrient agar, this study shows that a simple plate-based assay can detect a strong and repeatable land-cover signal in stormwater runoff. Shannon and Gini–Simpson morphotype diversity were consistently highest at the least impervious site and lowest at the most impervious site, whereas rainfall and temperature showed weak associations within the sampled range. The study does not capture the full runoff microbiome, but it does provide a clear culture-based observation that impervious cover is associated with runoff morphotype diversity across repeated storms.

## OBSERVATION

Urban land-cover changes what stormwater runoff mobilizes. Impervious surfaces increase runoff and are associated with poorer stormwater quality, whereas vegetated surfaces can reduce runoff and alter the material carried in flow (1–3). Previous studies have linked land use and impervious cover to stormwater microbial patterns (4, 5), but simple culture-based observations across repeated storms remain limited. Here, runoff from three Chapel Hill catchments spanning a strong impervious-cover gradient was sampled across 25 storms. Colonies grown on nutrient agar were grouped into visible morphotypes, and Shannon, Gini–Simpson, and Pielou evenness were calculated from morphotype area fractions. Higher culture-based morphotype diversity was expected at the less impervious catchments, with rainfall and temperature treated as secondary covariates.

### The study

All samples were collected in Chapel Hill, NC, at three catchments representing green space, residential space, and commercial space. Sampling coordinates were (35.980233, −78.999225) for Hollow Rock, (35.955356, −79.020751) for Springcrest, and (35.962457, −79.052690) for Timberlyne. Catchment polygons were delineated in Google Earth Engine from high-resolution aerial imagery, and land-cover percentages were calculated from classified pixels (6). Commercial space had the highest percentage of impervious cover, followed by residential and green space, whereas vegetation showed the opposite pattern (Fig. 1).

**Fig 1:**
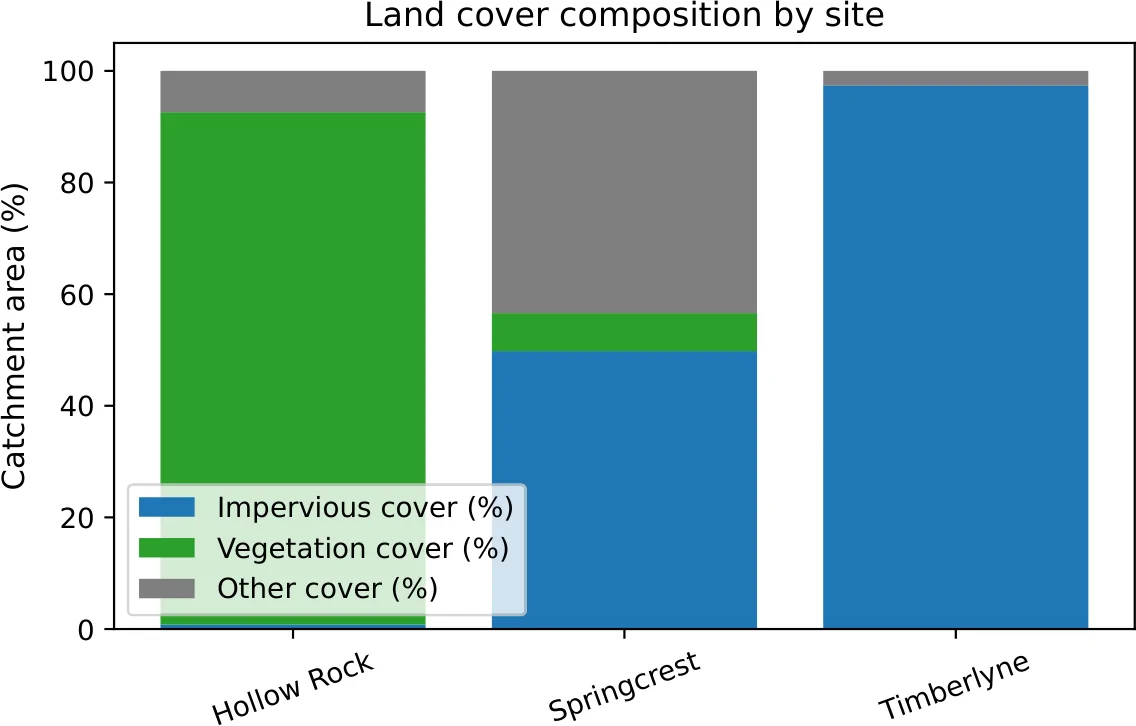
Land-cover composition by site. Mean percentages of impervious, vegetated, and other cover within each catchment are shown for the green space (Hollow Rock), residential space (Springcrest), and commercial space (Timberlyne) sites. Vegetation is shown in green.

Only qualifying storm events were sampled, defined as visible surface flow with at least 5 mm of precipitation. This yielded 75 site-storm samples across 25 storms. No dry-weather or non-storm controls were collected. Daily rainfall and mean temperature were obtained from the nearest NCEI station (7). At each site and storm, approximately 10 mL of runoff was collected into sterile bottles and plated the same day on nutrient agar. Plates were T-streaked, incubated at 37°C for 48 h, and photographed from above. Nutrient agar was used as a practical general heterotrophic medium for cross-sample comparison, but it recovers only a subset of runoff microorganisms.

Plate images were segmented into uncolonized agar and up to six visible morphotype classes based on colony color and texture. The workflow followed the logic of OpenCFU, whose algorithm was validated against human counts for accuracy and robustness in segmenting circular objects (8). Colony morphotypes have also been used as a preliminary way to distinguish different species or biotypes in environmental samples, although they underestimate total taxonomic diversity (9). The indices are therefore interpreted here as culture-based morphotype diversity, not total microbial diversity. Shannon, Gini–Simpson, and Pielou evenness were calculated from morphotype area fractions after excluding uncolonized agar.

For each index, a linear mixed-effect model was fit with land-use type, 24-h rainfall, and daily mean temperature as fixed effects and storm as a random intercept. Cross-validation folds were defined by storm.

### Results

Across 25 storms, culture-based morphotype diversity showed the same ordering in every main comparison. Shannon diversity and Gini–Simpson diversity were highest at the green catchment, intermediate at the residential catchment, and lowest at the commercial catchment, while evenness showed the opposite pattern (Fig. 2). Median Shannon diversity was about 1.05–1.07 at the green site, 0.89–0.90 at the residential site, and near 0.60 at the commercial site. Median Gini–Simpson diversity was about 0.61–0.62, 0.56, and 0.41, respectively. Median evenness was lowest at the green site, about 0.76, intermediate at the residential site, about 0.81–0.82, and highest at the commercial site, about 0.86–0.87. Because these indices were calculated from nutrient agar morphotype area fractions, they describe visible colony heterogeneity under the chosen culture conditions rather than the full runoff microbiome.

**Fig 2:**
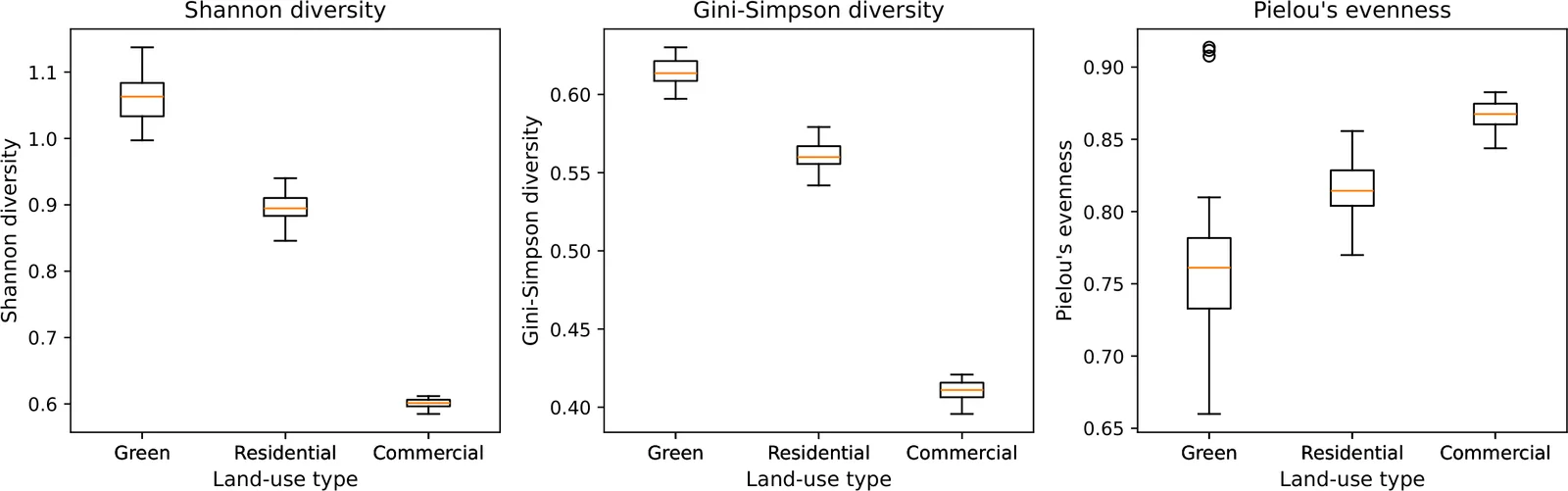
Distribution of alpha diversity indices by land-use type. Boxplots summarize Shannon diversity (left), Gini–Simpson diversity (center), and Pielou’s evenness (right) across 25 storms at each site.

Storm-level mixed-effect models reproduced the same pattern. For Shannon diversity, the green and residential catchments had strong positive coefficients relative to the commercial baseline (*P* < 0.001 for both; *β* = 0.461 and 0.295, respectively), while rainfall and temperature were not significant (*P* = 0.850 and *P* = 0.135; *β* = −0.00003 per mm and 0.00087 per °C, respectively). Gini–Simpson diversity showed the same result, with positive land-use coefficients for green and residential catchments (*P* < 0.001 for both; *β* = 0.204 and 0.150, respectively) and weak rainfall and temperature effects (*P* = 0.797 and *P* = 0.125; *β* = 0.000014 per mm and 0.00030 per °C, respectively). Evenness showed the reverse land-use pattern, with negative coefficients for green and residential catchments relative to commercial (*P* < 0.001 for both; *β* = −0.104 and −0.051, respectively) and again weak weather effects (*P* = 0.142 and *P* = 0.997; *β* = 0.00042 per mm and −0.000004 per °C, respectively). Conditional *R*² values were 0.983 for Shannon, 0.990 for Gini–Simpson, and 0.552 for evenness. Because folds were defined by storm rather than by site, the cross-validation results indicate within-study consistency across repeated storms, not predictive transfer to new catchments.

### Discussion

The main result was a repeatable impervious-cover signal in culture-based runoff morphotypes across storms. The least impervious catchment consistently yielded higher Shannon and Gini–Simpson diversity than the most impervious catchment, while evenness was highest at the commercial site. This is consistent with the idea that more vegetated catchments contribute a wider range of culturable morphotypes during storms, whereas heavily paved catchments contribute a narrower set. Biologically, the combination of higher Shannon and Gini–Simpson diversity but lower evenness at the green site is consistent with a broader culturable morphotype pool dominated by a few common colony types, whereas the commercial site is consistent with fewer visible morphotypes at more similar area fractions. These assay-level patterns do not establish taxonomic identities or mechanisms. Rainfall and temperature were weak within the sampled range once land use was included, suggesting that the catchment contrast dominated storm-to-storm weather variation in this data set.

That result is narrow. First, all samples were collected during storm events, so the data set cannot distinguish storm-specific mobilization from baseline between-catchment differences under dry conditions. Second, nutrient agar and the chosen incubation conditions recover only a subset of runoff microorganisms. Third, the image-based morphotype workflow was not benchmarked here against known mixtures, isolate identities, or sequencing-based community profiles. Finally, one catchment represented each land-use category, so land use and site are not separable in this data set. The study therefore supports a repeated three-catchment observation, not a general site-independent model of urban runoff morphotype diversity.

### Conclusion

Within this nutrient agar assay, impervious cover was strongly associated with runoff morphotype diversity across 25 storms. The finding is robust within the sampled catchments, but it should be interpreted as a culture-based observation rather than a measure of total microbial diversity.

## Data Availability

The data set supporting this study is available in Zenodo at https://doi.org/10.5281/zenodo.20386250. The deposited file is data_masterbook.xlsx.
